# CD5L Promotes M2 Macrophage Polarization through Autophagy-Mediated Upregulation of ID3

**DOI:** 10.3389/fimmu.2018.00480

**Published:** 2018-03-12

**Authors:** Lucía Sanjurjo, Gemma Aran, Érica Téllez, Núria Amézaga, Carolina Armengol, Daniel López, Clara Prats, Maria-Rosa Sarrias

**Affiliations:** ^1^Innate Immunity Group, Germans Trias i Pujol Health Sciences Research Institute (IGTP), Barcelona, Spain; ^2^Network for Biomedical Research in Diabetes and Associated Metabolic Disorders (CIBERDEM), Barcelona, Spain; ^3^Network for Biomedical Research in Hepatic and Digestive Diseases (CIBERehd), Barcelona, Spain; ^4^Childhood Liver Oncology Group, Program of Predictive and Personalized Medicine of Cancer (PMPCC), Germans Trias i Pujol Health Sciences Research Institute (IGTP), Badalona, Spain; ^5^Departament de Física, Escola Superior d’Agricultura de Barcelona, Universitat Politècnica de Catalunya – BarcelonaTech Castelldefels, Barcelona, Spain

**Keywords:** CD5L, autophagy, macrophage polarization, scavenger receptor cysteine rich, mathematical algorithm, ID3, phagocytosis, efferocytosis

## Abstract

CD5L (CD5 molecule-like) is a secreted glycoprotein that controls key mechanisms in inflammatory responses, with involvement in processes such as infection, atherosclerosis, and cancer. In macrophages, CD5L promotes an anti-inflammatory cytokine profile in response to TLR activation. In the present study, we questioned whether CD5L is able to influence human macrophage plasticity, and drive its polarization toward any specific phenotype. We compared CD5L-induced phenotypic and functional changes to those caused by IFN/LPS, IL4, and IL10 in human monocytes. Phenotypic markers were quantified by RT-qPCR and flow cytometry, and a mathematical algorithm was built for their analysis. Moreover, we compared ROS production, phagocytic capacity, and inflammatory responses to LPS. CD5L drove cells toward a polarization similar to that induced by IL10. Furthermore, IL10- and CD5L-treated macrophages showed increased LC3-II content and colocalization with acidic compartments, thereby pointing to the enhancement of autophagy-dependent processes. Accordingly, siRNA targeting ATG7 in THP1 cells blocked CD5L-induced CD163 and Mer tyrosine kinase mRNA and efferocytosis. In these cells, gene expression profiling and validation indicated the upregulation of the transcription factor ID3 by CD5L through ATG7. In agreement, ID3 silencing reversed polarization by CD5L. Our data point to a significant contribution of CD5L-mediated autophagy to the induction of ID3 and provide the first evidence that CD5L drives macrophage polarization.

## Introduction

Macrophages are innate immune cells present in all vertebrate tissues. To ensure homeostasis, these cells respond to internal and external cues and exert trophic, regulatory, repair, and effector functions (1). However, they are also involved in the pathogenesis of major human diseases, ranging from infections, atherosclerosis, chronic inflammatory diseases including arthritis and diabetes, degenerative conditions such as Alzheimer’s disease, and cancer (2).

The functional diversity of macrophages can be attributed to their ability to alter their phenotype in response to changes in the microenvironment (3). This plasticity allows them to acquire a wide range of functions, from proinflammatory, pathogen-eliminating, and subsequent tissue-damaging (referred to as M1 or classically activated macrophages) to anti-inflammatory, immunosuppressive, and wound-healing (referred to as M2 or alternatively activated) (4). In this context and given the inherent plasticity of these cells, there is growing interest in applying knowledge of their polarization to treat human diseases. In this regard, the repolarization of macrophages might offer an attractive therapeutic approach in diseases such as cancer (5). Various subpopulations of polarized macrophages have been defined on the basis of their *in vitro* stimulation. M1 prototypic macrophages are induced by Th1 inflammatory cytokines, microbial factors, or a combination of the two. In turn, the M2 subset comprises macrophages induced by exposure to Th2 cytokines IL4 and IL13, immune complexes in combination with IL1β or LPS, glucocorticoids, anti-inflammatory cytokines IL10 and TGFβ, or tumor microenvironmental factors such as IL6 and leukemia inhibitor factor (6, 7). Here, we adopted the macrophage nomenclature proposed by Murray et al. based on the activation stimulus, i.e., M-INF/LPS, M-IL4, and M-IL10, as well as M-dexamethasone (DXM), which have also been referred to as M1, M2a, and M2c, respectively (8). These macrophage subsets have been classified on the basis of their gene signatures, activation signaling pathways, surface molecule expression pattern, secretory profile, and functional properties (6, 7, 9–12). However, in this regard, most studies have been performed in murine models. Although these models have led to great advances, they show important discrepancies with the human. Moreover, information regarding human macrophage polarization is limited and scattered, especially regarding functional characterization. These observations thus highlight the urgent need for further advancement of our knowledge of human macrophage polarization (5).

Macrophages are the main source of CD5-like protein (CD5L), a 40-kDa soluble glycoprotein that belongs to the scavenger receptor cysteine rich superfamily (13). CD5L is involved in a broad spectrum of biological functions (14). Various mouse models of disease support the notion that CD5L participates in the pathogenesis of inflammatory processes, including cancer, by preventing the apoptosis of macrophages and other cell types (15–20). Human CD5L has also been shown to modulate other aspects of macrophage biology, namely anti-microbial responses to *Mycobacterium tuberculosis* (21) and TLR activation through increased autophagic mechanisms (22, 23). Moreover, CD5L contributes to atherogenesis by promoting oxLDL uptake and to macrophage-endothelial cell adhesion (24).

CD5L circulates in serum in relatively high amounts (25), and results of proteomic profiling highlight it as a putative serum biomarker for inflammatory conditions such as atopic dermatitis (26), Kawasaky disease as well as liver cirrhosis (27–29). These significant alterations of plasma CD5L levels, together with the reports on its control of macrophage responses, have consistently suggested a functional role of this protein in host inflammatory reactions.

Like for many key modulators of macrophage activity, CD5L expression is tightly regulated in cells and tissues (20, 30, 31), being upregulated under inflammatory conditions and also during cardiovascular and metabolic pathologies. Likewise, *in vitro* cultured macrophages do not express CD5L unless they have been previously activated with specific stimuli (19, 24, 32).

Here, we studied the involvement of CD5L in human macrophage polarization. To this end, we performed a comprehensive analysis of human macrophages polarized *in vitro*. Using a novel mathematical algorithm to analyze phenotypic changes, together with functional studies, we reveal for the first time that—like IL10—CD5L drives macrophages toward an M2 phenotype. In addition, CD5L expression was restricted to those macrophages treated with IL10. Furthermore, our results provide the first evidence that CD5L involvement in M2 macrophage polarization is dependent on autophagic mechanisms and ID3 transcription factor.

## Materials and Methods

### Primary Cells and Cell Lines

All studies involving human samples were conducted following the Declaration of Helsinki principles and current legislation on the confidentiality of personal data and were approved by the Human Ethics Committee of the *Hospital Universitari Germans Trias i Pujol*. Buffy coats, provided by the Blood and Tissue Bank (Barcelona, Spain), were obtained from healthy blood donors following the institutional standard operating procedures for blood donation and processing, including informed consent. CD3^+^ cells were depleted by RosetteSep human CD3 depletion cocktail (StemCell Technologies). Peripheral blood mononuclear cells (PBMC) were isolated as described previously (23) by Ficoll-Paque (GE Healthcare) density gradient centrifugation at 400 × *g* for 25 min. Recovered cells were washed twice in PBS and counted using Perfect-Count microspheres (Cytognos), following the manufacturer’s instructions. Peripheral blood monocytes (PB monocytes) were isolated by adherence in a 5% CO_2_ incubator at 37°C in RPMI-1640 2 mM glutamine (Lonza) supplemented with 10% heat-inactivated human AB serum (Sigma-Aldrich) for 30 min. Non-adherent cells were removed and adherent cells were washed twice with PBS and incubated in RPMI-1640 2 mM glutamine, 10% heat-inactivated fetal bovine serum (FBS, Lonza), 100 U/mL penicillin, and 100 µg/mL streptomycin (Sigma-Aldrich) for 24 h prior to the experiments. The percentage of adherent CD14^+^ cells (PB monocytes) routinely obtained was 94.98% (±3.26%). In phagocytosis assays, PB monocytes were differentiated by incubation in RPMI 10% heat-inactivated FBS for 7 days prior to the experiments, as described previously (24).

Stably transfected THP1-vector and THP1-CD5L cell lines were generated as described in Amézaga et al. (24). Cells were grown in culture medium (RPMI-1640 2 mM glutamine, 100 U/mL penicillin, and 100 µg/mL streptomycin) supplemented with 10% heat-inactivated FBS and 250 µg/mL geneticin (Gibco). Prior to the experiments, cells were differentiated to macrophages by incubation with 10 ng/mL of phorbol 12-myristate 13-acetate (PMA, Sigma-Aldrich) in culture medium for 24 h. They were then washed with PBS and grown in culture medium for 24 h. These cells are referred to as THP1. HepG2 cells were purchased from ATCC (The American Type Culture Collection) and cultured in EMEM supplemented with 2 mM glutamine (Lonza), 100 U/mL penicillin, 100 µg/mL streptomycin, and 10% heat-inactivated FBS.

### *In Vitro* Polarization of Macrophages

PB monocytes and THP1 macrophages were polarized by incubation during the indicated times with 50 ng/mL IFNγ (Preprotech) plus 100 ng/mL LPS from *Escherichia coli* O111:B4 (Sigma-Aldrich) (INF/LPS), 40 ng/mL IL4 (Preprotech), 50 ng/mL IL10 (Preprotech), or 40 ng/mL DXM (Kern pharma). The control population was incubated in culture medium (−) without polarizing cytokines. To assess the effect of human CD5L (*Homo sapiens* CD5L, hsCD5L) on PB monocytes, these cells were incubated with 1 µg/mL albumin (Alb) purified from human plasma (Grifols), which was used as control protein, or 1 µg/mL endotoxin-free recombinant CD5L (rCD5L) expressed in Chinese Hamster Ovary cells, as detailed in Ref. (24). As a positive control of CD5L mRNA upregulation, cells were treated with 1 µM T1317 (Tocris Bioscience, Bristol, UK), plus 1 µM 9cRa (Sigma-Aldrich).

### Multicolor Flow Cytometry Analysis

PB monocytes (10^6^ cells/well) were plated in six-well plates and incubated for 72 h with the polarizing stimuli at a final concentration of 5% FBS. They were then detached with accutase (Sigma-Aldrich), washed in PBS, and incubated with 100 µL of blocking buffer [PBS containing 10% human AB serum (Sigma-Aldrich), 2% FCS (Lonza), and 0.02% NaN_3_ (Sigma-Aldrich)]. Cells were then labeled in brilliant stain buffer (BD Bioscience) with a combination of fluorescently conjugated monoclonal antibodies against HLADR, CD80, CD23, CD206, and CD163 (BD Biosciences). Flow cytometry analysis was performed on a BD LSRFortessa instrument using FACSDiva software (BD Biosciences), with 10,000 events acquired for each sample. Integrated median fluorescence intensity (iMFI) was computed by multiplying the relative frequency (percentage of positive) of cells expressing each marker by the median fluorescence intensity (MFI) of the cell population.

### Algorithm Development for the Classification of Polarized Macrophages on the Basis of Their Phenotypic Responses to IFN/LPS, IL4, or IL10

For each donor *d* (*d* = 1:26), we defined a vector that included the iMFI measurements of the five surface markers under a certain stimulus st (st = IFN/LPS, IL4, or IL10)

(1) $${\text{iMFI}}_{\text{d},\text{st}}={\left({\text{iMFI}}_{\text{HLADR}},{\text{iMFI}}_{\text{CD80}},{\text{iMFI}}_{\text{CD23}},{\text{iMFI}}_{\text{CD206}},{\text{iMFI}}_{\text{CD163}}\right)}_{d,\text{st}}$$

The mean response of all the samples to a specific stimulus was then calculated and written in the corresponding vector. Therefore, for each stimulus we obtained one mean vector, containing the five mean values of iMFI measurements, one for each surface marker.

(2) $$\begin{array}{l} {\text{iMFI}}_{\text{mean},\text{st}}=(\text{mean}\left\{{\text{iMFI}}_{\text{HLADR},d}\right\},\text{mean}\left\{{\text{iMFI}}_{\text{CD80},d}\right\},\\ \text{ mean}\left\{{\text{iMFI}}_{\text{CD23},d}\right\},\text{mean}\left\{{\text{iMFI}}_{\text{CD2016},d}\right\},\text{mean}\left\{{\text{iMFI}}_{\text{CD163},d}\right\}{)}_{\text{st}} \end{array}$$

Given that (i) the presence of surface markers on the membrane and the resulting fluorescence intensity scale may differ between markers and that (ii) we sought to simply determine the tendency of the surface marker to increase or decrease under a certain stimulus, we defined a normalized scale of iMFI for each of the markers, thus obtaining the *normalized iMFI*, $\hat{\text{iMFI}}$. To carry out this normalization, we took as maximum reference values for each surface marker those arising from the five mean vectors, ${\text{iMFI}}_{\text{mean},\text{st}}$. The normalization was then applied to all individual measured values (i.e., for each donor, stimulus, and surface marker) as follows, fixing at 1 those values that exceeded it:

(3) $$\begin{array}{l} {\hat{\text{iMFI}}}_{\text{sm},d}=\text{min}\left\{\frac{{\text{iMFI}}_{\text{sm},d}}{\text{max}{\left\{{\text{iMFI}}_{\text{sm,mean,}}\right\}}_{\text{st}=\text{IFN}/\text{LPS},\text{IL4 or IL1}0}};1\right\};\\ \text{ sm}=\text{HLADR, CD80, CD23, CD206, or CD163} \end{array}$$

**Figure 1:**
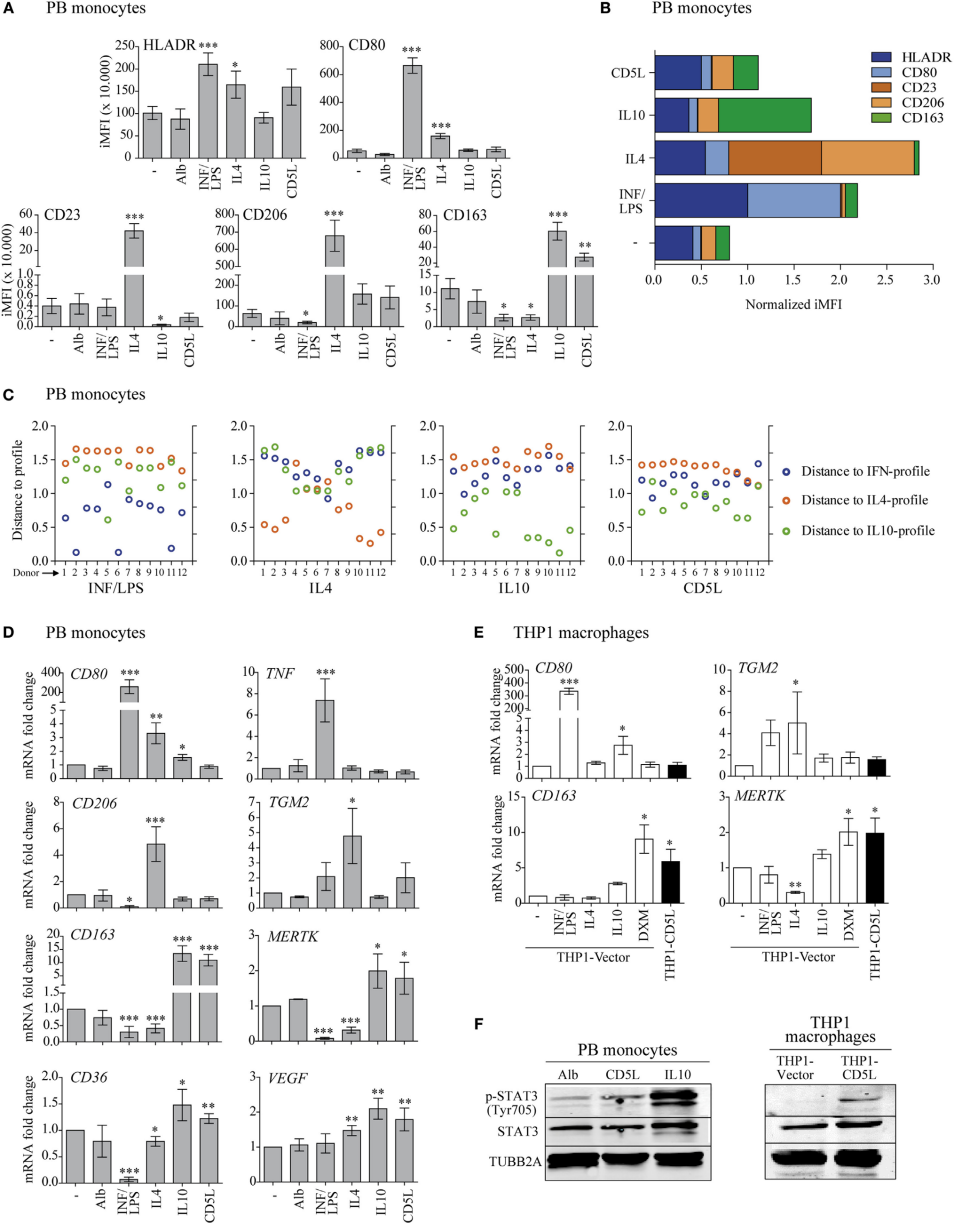
CD5L induces a phenotype in macrophages like IL10. **(A)** Multicolor flow cytometry analysis of HLADR, CD80, CD23, CD206, and CD163 marker profiles in PB monocytes treated for 72 h with medium alone (−), standard polarization stimuli (INF/LPS, IL4, and IL10), recombinant CD5L (CD5L), or albumin (Alb) from human serum. The graphs show the integrated median fluorescence intensity (iMFI) values of each maker. Data from 28 and 12 blood donors for standard stimuli or rCD5L/Alb are included, respectively. **(B)** Normalized profiles. Mean response patterns of monocytes to stimuli, represented by the normalized vectors ${\hat{\text{iMFI}}}_{\text{pattern},\text{st}}$ (st = M, IFN/LPS, IL4, IL10, and CD5L). Standard stimuli: *n* = 26, rCD5L/Alb *n* = 12. **(C)** Distance of sample response to normalized profiles. Blue: distance to IFN/LPS pattern (dist_st–IFN/LPS,_ *_d_*); orange: distance to IL4 pattern (dist_st–Il4,_ *_d_*); green: distance to IL10 pattern (dist_st–Il10,_*_d_*). Samples from 12 blood donors stimulated with IFN/LPS, IL4, IL10, or CD5L are included. **(D)** PB monocytes were treated for 24 h, and the amount of mRNA encoding *CD80, TNF, CD206, TGM2, CD163*, Mer tyrosine kinase (*MERTK*), *CD36*, and vascular endothelial growth factor (*VEGF*) was measured by RT-qPCR. Data show mean of at least four blood donors. **(E)** THP1 macrophages were incubated with the indicated stimuli for 24 h, and the amount of mRNA encoding *CD80, TGM2, CD163*, and *MERTK* was measured by RT-qPCR. Data show mean of at least three independent experiments. **(F)** Western blot images of PB monocytes incubated for 24 h with human albumin (Alb), rCD5L or IL10 (left), or THP1-vector, and THP1-CD5L macrophages (right) probed with specific antibodies against phosphorylated STAT3 (Tyr705), STAT3, and TUBB2A. Representative blot from three independent experiments. In **(A,D,E)**, data are presented as mean ± SEM. *P ≤ 0.05; **P ≤ 0.01; ***P ≤ 0.001 Student’s *t*-test, vs. macrophages cultured in medium alone (−) column.

This normalization algorithm was also applied to the mean vectors, thus obtaining the dimensionless mean vectors to be used as response patterns to the stimuli, ${\hat{\text{iMFI}}}_{\text{pattern},\text{st}}$, which are shown in Figure 1B.

(4) $$\begin{array}{l} {\hat{\text{iMFI}}}_{\text{pattern},\text{st}}\text{=}({\hat{\text{iMFI}}}_{\text{HLADR, mean}}\text{,}{\hat{\text{iMFI}}}_{\text{CD80, mean}}\text{,}{\hat{\text{iMFI}}}_{\text{CD23, mean}}\text{,}\\ {\hat{\text{iMFI}}}_{\text{CD206, mean}}\text{,}{\hat{\text{iMFI}}}_{\text{CD163, mean}}{)}_{\text{st}}. \end{array}$$

The response pattern was used to classify sample response to a given stimulus. It is hypothesized that a sample stimulated with IFN/LPS will show the lowest distance to the IFN/LPS pattern $\left({\hat{\text{iMFI}}}_{\text{pattern},\text{IFN}/\text{LPS}}\right)$ when its response is also compared with the IL4 pattern $\left({\hat{\text{iMFI}}}_{\text{pattern},\text{IL}4}\right)$ and IL10 pattern ${\hat{(\text{iMFI}}}_{\text{pattern},\text{IL}10}\text{)}$.

Let us assume that we have a sample of a certain donor, *d*, that has been treated with the stimulus st (st = IFN/LPS, IL4, or IL10). This sample is then experimentally analyzed and its surface markers are determined. The normalized concentration of each surface marker is evaluated through the vector ${\hat{\text{iMFI}}}_{\text{st},\text{d}}\text{=}{\left({\hat{\text{iMFI}}}_{\text{HLADR, }d}\text{, }{\hat{\text{iMFI}}}_{\text{CD80, }d}\text{, }{\hat{\text{iMFI}}}_{\text{CD23, }d}\text{, }{\hat{\text{iMFI}}}_{\text{CD206, }d}\text{,}{\hat{\text{iMFI}}}_{\text{CD163, }d}\right)}_{\text{st}}$. This magnitude set will be compared with the three patterns by using three geometrical distances between vectors, namely dist_st–IFN/LPS,_ *_d_*, dist_st–IL4,_ *_d_*, and dist_st–IL10,_ *_d_*, as follows:

(5) $${\text{dist}}_{\text{st}-\text{IFN/LPS},d}=\left|{\hat{\text{iMFI}}}_{\text{st},\text{d}}-{\hat{\text{iMFI}}}_{\text{pattern},\text{IFN}/\text{LPS}}\right|$$

(6) $${\text{dist}}_{\text{st}-\text{IL}4,d}=\left|{\hat{\text{iMFI}}}_{\text{st},\text{d}}-{\hat{\text{iMFI}}}_{\text{pattern},\text{IL}4}\right|$$

(7) $${\text{dist}}_{\text{st}-\text{IL}10,d}=\left|{\hat{\text{iMFI}}}_{\text{st},\text{d}}-{\hat{\text{iMFI}}}_{\text{pattern},\text{IL}10}\right|$$

The sample is classified on the basis of the minimum distance, i.e., when the sample was stimulated with IFN/LPS, we expect that $\text{min}\left\{{\text{dist}}_{\text{st}-\text{IFN}/\text{LPS},d},{\text{dist}}_{\text{st}-\text{IL}4,d},{\text{dist}}_{\text{st}-\text{IL}10,d}\right\}={\text{dist}}_{\text{st}-\text{IFN/LPS},d}$.

The same procedure was applied to assess the distances between the response of cells to CD5L and the three patterns (dist_CD5L–IFN/LPS,_ *_d_*, dist_CD5L–IL4,_ *_d_*, and dist_CD5L–IL10,_ *_d_*), once the normalized ${\hat{\text{iMFI}}}_{\text{CD}5\text{L},\text{d}}$ had been obtained by means of Eq. 3 (*d* = 1:12). The values of all distances are shown in Figure 1C.

The complete algorithm for classifying the stimulated samples is summarized in Table 1.

**Table 1:** Algorithm for classifying a sample *n* treated with an unknown stimulus.

(1) Experimental determination of the ${\text{iMFI}}_{\text{n}}$ vector of the stimulated sample (*n*) by means of flow cytometry, including the measurements of the five surface markers $\left({\text{iMFI}}_{\text{HLADR.}n}{\text{, iMFI}}_{\text{CD80.}n}{\text{,iMFI}}_{\text{CD23.}n}{\text{, iMFI}}_{\text{CD206.}n}{\text{, iMFI}}_{\text{CD163.}n}\right)$ (Eq. 1).
(2) Evaluation of the normalized concentration vector, ${\hat{\text{iMFI}}}_{\text{n}}$, with the normalization factors given by Eq. 3.
(3) Evaluation of the geometrical distance between ${\hat{\text{iMFI}}}_{\text{n}}$ and each of the pattern vectors ${\hat{(\text{iMFI}}}_{\text{pattern},\text{IFN}/\text{LPS}}\text{, }{\hat{\text{iMFI}}}_{\text{pattern},\text{IL}4}\text{, }{\hat{\text{iMFI}}}_{\text{pattern},\text{IL}10}\text{)}$ (Eqs 5–7).
(4) Classification of the sample according to the minimum distance criterion: $\text{min}\left\{{\text{dist}}_{n-\text{IFN/LPS}},{\text{dist}}_{n-\text{IL4}},{\text{dist}}_{n-\text{IL}10}\right\}$.

### RNA Extraction and Quantitative RT-PCR

PB monocytes or THP1 macrophages (1 × 10^6^ cells/well) were incubated for 24 h in RPMI medium containing 5% FCS and the polarizing stimuli. Cells were then washed with PBS and disrupted with QIAzol Lysis Reagent (Qiagen), and RNA was extracted using the miRNeasy Mini Kit (Qiagen). Total RNA (1 µg) was reverse transcribed using the RNA to cDNA EcoDry™ Premix (Clontech). Each RT reaction was then amplified in a LightCycler^®^ 480 PCR system (Roche) using the KAPA SYBR Fast Master Mix (KAPA Biosystems). Samples were incubated for an initial denaturation at 95°C for 5 min, then 40 PCR cycles were performed using the following conditions: 95°C for 10 s, 60°C for 20 s, and 72°C for 10 s. The primer pairs used in the study are listed in Table 2. Gene expression values were normalized to the expression levels of *GAPDH* (glyceraldehyde 3-phosphate dehydrogenase). Fold induction levels were calculated by using the expression level of each gene in untreated conditions (−) as a reference.

**Table 2:** List of primers used in this study.

Gene	Forward primer (5′ → 3′)	Reverse primer (5′ → 3′)
*CD80*	CTGCCTGACCTACTGCTTTG	GGCGTACACTTTCCCTTCTC
*TNF*	GAGGAGGCGCTCCCCAAGAAG	GTGAGGAGCACATGGGTGGAG
*TGM2*	CCTCGTGGAGCCAGTTATCAA	GTCTGGGATCTCCACCGTCTTC
*CD206*	ACACAAACTGGGGGAAAGGTT	TCAAGGAAGGGTCGGATCG
*CD163*	CACCAGTTCTCTTGGAGGAACA	TTTCACTTCCACTCTCCCGC
*MERTK*	CTCTGGCGTAGAGCTATCACT	AGGCTGGGTTGGTGAAAACA
*CD36*	GAGAACTGTTATGGGGCTAT	TTCAACTGGAGAGGCAAAGG
*VEGF*	AGGGCAGAATCATCACGAAGT	AGGGTCTCGATTGGATGGCA
*CD5L*	GACGAGAAGCAACCCTTCAG	CCCAGAGCAGAGGTTGTCTC
*ATG7*	ATGATCCCTGTAACTTAGCCCA	CACGGAAGCAAACAACTTCAAC
*BEX1*	AGGCCCAGGAGTAATGGAGT	AACCGCCTACGATTTCCTCT
*ID3*	GAGAGGCACTCAGCTTAGCC	TCCTTTTGTCGTTGGACATGAC
*GAPDH*	TCTTCTTTTGCGTCGCCAG	AGCCCCAGCCTTCTCCA

### Measurement of Cytokine Secretion

PB monocytes (10^5^ cells/well) were incubated for 72 h with polarizing stimuli diluted in culture medium containing 5% FCS and subsequently stimulated with LPS (from *E. coli* 0111: B4, Sigma-Aldrich). After 4 h (for TNF) or 24 h for (IL1β and IL6), culture supernatant fractions were collected and assayed for cytokine production with OptEIA ELISA, following the manufacturer’s instructions (BD Biosciences). In experiments using THP1 macrophages treated with siRNA targeting *ID3* expression, cells were siRNA-transfected and allowed to differentiate (see below). They were then stimulated with LPS (5 × 10^4^ cells/well) and assayed for cytokine production as above. Color was developed by adding 3,3′,5,5′-tetramethylbenzidine liquid substrate (Sigma-Aldrich). Optical density was read at 450 nm on a Varioskan Flash microplate reader (ThermoFisher Scientific Inc.).

### ROS Measurement

PB monocytes (10^5^ cells/well) were polarized with the indicated stimuli for 72 h and then loaded with 10 µM dichloro-dihydro-fluorescein diacetate (H2-DCF-DA) (Sigma-Aldrich) in PBS for 30 min at 37°C in the dark. They were then washed twice and resuspended in 100 µL of PBS. Absorbance at 485 nm was measured using a Varioskan Flash microplate reader (ThermoFisher Scientific Inc.). Intracellular ROS levels were calculated by using the levels of control cells (−) as a reference.

### Analysis of Phagocytosis by Flow Cytometry

2.5 × 10^5^ PB monocytes or THP1 macrophages were plated in 24-well plates, polarized for 72 h and incubated with 3 µM YG Fluoresbrite^®^ microspheres (Polysciences), *E. coli* K-12, or *Staphylococcus aureus* fluorescent bioparticles (Molecular Probes) at the indicated ratios (macrophage: microsphere/bacteria) for 1 h at 37 or 4°C. Incubation at 4°C was performed to measure extracellular attachment rather than internalization, since no uptake occurs at this temperature. Cells were then harvested by a 20-min incubation with accutase (Sigma-Aldrich). They were then extensively washed with cold PBS and fixed with PBS containing 4% paraformaldehyde (Panreac) for 30 min. Phagocytosis was quantified by flow cytometry on a FACSCantoII instrument (BD Bioscience) using the FACSDiva software (BD Bioscience).

### Induction of Apoptosis and Efferocytosis Assay

1 × 10^6^ HepG2 cells were plated in six-well plates and labeled with 5 µM CellTrace CFSE (Invitrogen) in PBS for 20 min at 37°C in 5% CO_2_. They were then washed and medium was replaced by EMEM 10% FBS. Twenty-four hours later, apoptosis was induced by 45 min UV irradiation followed by 1 h resting at 37°C. Then 3,3’-dihexyloxacarbocyanine iodide (DIOC, Sigma-Aldrich) and propidium iodide (PI; Sigma-Aldrich) were used to measure apoptosis by flow cytometry, obtaining 16.2% ± 8.1% live cells (DIOC+, PI−), 9.4% ± 5.0% early apoptotic cells (DIOC−, PI−), and 68.3% ± 5.2% late apoptotic cells and necrotic cells (PI+). An efferocytosis assay was performed by co-culturing THP1 cells pre-labeled, as above with 0.5 µM CellTracker™ Deep Red Dye (Thermo Fisher), with CFSE-labeled apoptotic HepG2 cells for 1 h at the indicated ratios (macrophage: apoptotic cell) and at 37 or 4°C. Cells were then harvested by a 20-min incubation with accutase (Sigma-Aldrich), extensively washed with cold PBS, and fixed. Flow cytometry was performed on a FACSCantoII instrument. FACSDiva software was used to quantify percentages of Deep Red-labeled macrophages that phagocytosed CFSE-labeled apoptotic cells. In addition, efferocytosis was observed by fluorescent microscopy using an Axio Observer Z1 DUO LSM 710 confocal system (Carl Zeiss Microscopy GmbH).

### Western Blot Analysis of Cell Lysates

For STAT3 phosphorylation, LC3 conversion and ID3 detection analysis, PB monocytes or THP1 macrophages (1 × 10^6^ cells/well) were plated in six-well plates and polarized by incubation with the indicated stimuli at 37°C for the indicated periods. They were then washed in cold TBS and lysed in TBS lysis buffer [20 mM Tris, pH 7.5, containing 150 mM NaCl, 1 mM EDTA, 1 mM EGTA, 1% Triton X-100, 1 mM Na^3^VO^4^, 1 mM PMSF, and complete protease inhibitor cocktail (all from Sigma-Aldrich)] for 30 min at 4°C. For STAT3 and LC3, nuclei and cell debris were removed by centrifugation at 8,000 × *g* for 15 min, while for ID3, total cell lysates were resolved. Protein concentration was measured with the BCA protein assay reagent kit (Thermo Fisher Scientific), following the manufacturer’s instructions. To this end, 40–50 µg of protein from cell lysates were resolved in 10% SDS-polyacrylamide gels (12% for LC3) under reducing conditions and electrophoretically transferred to nitrocellulose membranes (Bio-Rad Laboratories). These were then blocked with Starting Block TBS buffer (Thermo Fisher) for 1 h at room temperature and incubated overnight at 4°C with mAb anti-phosphorylated STAT3 Tyr705 (Clone 9E12, 05-485, Millipore), poAb anti-STAT3 (06-596, Millipore), poAb anti-LC3 (NB100–2220, Novus Biologicals), and mAb anti-ID3 (9837, Cell Signaling Technology) diluted in blocking buffer. Blots were also probed against beta-tubulin (mAb anti-TUBB2A, T9026, Sigma-Aldrich) or Histone H3 (poAb anti-HIST3H3, 9715, Cell Signaling Technology) to determine equal loading. The membranes were subsequently incubated for 60 min at room temperature with the appropriate fluorescently coupled secondary antibodies (IRDye680Cw-conjugated goat anti-rabbit IgG or IRDye 800Cw-conjugated goat anti-mouse IgG, LI-COR Biosciences, 926–32,221 and 926–32,210, respectively) diluted in blocking buffer. Three 15-min washes between steps were performed with TBS-0.01% Tween 20 (Merck Millipore). Bound antibody was detected with an Odyssey Infrared Imager (LI-COR), and densitometric analysis was performed using the Odyssey V.3 software (LI-COR).

### Fluorescence Microscopy Studies

PB monocytes (10^5^ cells/well) were plated and incubated with the indicated stimuli for 72 h on Millicell EZ slides (Merck Millipore). Cells were fixed with PBS containing 4% paraformaldehyde (Panreac) and incubated for 24 h at 4°C with moAb anti-CD5L (Abnova) or poAb anti-LC3 (Novus Biologicals) in PBS containing 0.3% Triton X-100 and 10% human AB serum (Sigma-Aldrich). Cells were subsequently incubated for 1 h at room temperature with Alexa Fluor^®^ 488 F(ab’)_2_ fragment of goat anti-mouse IgG or Alexa Fluor^®^ 647 F(ab’)_2_ fragment of goat anti-rabbit IgG (Molecular Probes) in PBS containing 0.3% Triton X-100. Between steps, unbound antibodies were removed with three washes with PBS. Finally, nuclei were stained for 10 min at room temperature with PBS containing 800 nM Hoechst 33,258 solution (Sigma-Aldrich). Cells were then washed three times with PBS, and coverslips were mounted in Fluoromount media (Sigma-Aldrich) and left at 4°C overnight. To determine autophagic flux, culture medium was replaced by prewarmed RPMI containing 100 nM LysoTracker Red (Molecular Probes), and cells were incubated at 37°C for 1 h before fixation. The slides were examined under an Axio Observer Z1 DUO LSM 710 confocal system and analyzed with ZEN Black software (Carl Zeiss Microscopy GmbH). LC3 and LC3-LysoTracker Red colocalized puncta per cell were determined using a green and red puncta colocalization macro and ImageJ software in threshold images with sizes from 3 to 30 pixel^2^ and puncta circularity 0.8–1, as described previously (23).

### Silencing of *CD5L, ATG7*, and *ID3* Expression

Undifferentiated THP1 cells were transfected with 10 nM of a set of four siRNAs targeting *CD5L, ATG7, ID3* or an equal concentration of a non-targeting negative control pool (ON-TARGET plus siRNA, Dharmacon) using INTERFERin (Polyplus-transfection SA), as previously described (23), following the manufacturer’s instructions. After 24 h, medium was replaced, and cells were differentiated for 24 h with culture medium supplemented with 10 ng/mL PMA. Next, this medium was replaced by culture medium for a further 24 h before being tested for *CD5L, ATG7*, or *ID3* expression by PCR or western blot and used in the functional assays described above. When indicated, cells were treated with 40 ng/mL DXM for an additional 24 h prior to analysis.

### Gene Expression Profiling Analysis

Total RNA was isolated and purified from 10^6^ THP1-vector or THP1-CD5L macrophages by using TRIzol reagent (Invitrogen) and RNeasy columns (Qiagen). cRNA was generated from 10 µg of total RNA by using superscript (Invitrogen) and the MessageAmp II-Biotin (Ambion, USA) RNA transcription-labeling kit (Enzo Biochem) cRNA was hybridized to the CodeLink™ Human Whole Genome Bioarray (Applied microarrays) at 37°C for 16 h by using the TrayMix hybridizer (BioTray, France). Samples were labeled with Cy5 (Bionova), and arrays were scanned with an InnoScan 700 scanner (Innopsys). Data normalization and analysis were performed with Bioconductor R, LIMMA package by the bioinformatics platform of CIBERehd, Spain. The microarray data have been deposited in the NCBI’s Gene Expression Omnibus database(https://www.ncbi.nlm.nih.gov/geo/) under accession number GSE111315. Lists of genes obtained from microarray analysis were associated with biological process annotations, as defined by the Gene Ontology (GO) Consortium (18). DAVID bioinformatics resources (19, 20) were used to search for statistically significant enrichment of functional categories.

### Statistical Analysis

Data are presented as mean ± SEM of at least three experiments. Student’s *t*-test was performed with Graphpad Prism V.5 software. Values of *P* ≤ 0.05 were considered significant.

## Results

### Polarization with CD5L Promotes an M2 Phenotypic Profile Like That Induced by IL10

To gain a thorough understanding of the role of CD5L in human macrophage polarization, we examined the phenotypic and also functional changes induced by this protein, when compared with the response to culture medium (−) or the standard polarization stimuli IFN/LPS, IL4, or IL10. Neither human PB monocytes nor THP1 macrophages express detectable levels of CD5L protein. Therefore, we supplemented PB monocyte cultures with human rCD5L or human Alb as control. Although the latter is not an inert protein, we have previously used it as negative control, observing no significant effects on PB monocytes in our assays. On the other hand, we generated a macrophage cell line that stably expresses human CD5L, referred to as THP1-CD5L (21, 24).

The initial analysis included combined flow cytometry studies of HLADR, CD80, CD206, CD23, and CD163 polarization markers on PB monocytes from healthy blood donors after treatment with medium alone, the standard polarization stimuli, or rCD5L (Figure 1A). In these experiments, INF/LPS selectively increased HLADR and CD80 and diminished CD206 and CD163 when compared with medium alone; IL4 increased HLADR, CD80, CD23, and CD206 and inhibited CD163; and IL10, as well as rCD5L, increased CD163, and reduced CD23. Collectively, these flow cytometry data were used to build an algorithm for macrophage polarization classification, thus facilitating the study of response patterns in an unbiased manner (Table 1). To this end, flow cytometry data on marker expression were normalized (Figure 1B). For each donor, the response was then compared with the normalized profiles of IFN/LPS, IL4, and IL10 by calculating the distance of each response to each of the standard stimuli. The shortest distance was considered optimal. By using this “minimum distance criterion,” 92% of the samples treated with IFN/LPS, IL4, or IL10 were correctly classified according to the applied stimuli (IFN/LPS, IL4, or IL10) (Figure 1C). The classification algorithm was then used to compare the distances between rCD5L-induced surface marker levels $\left({\hat{\text{iMFI}}}_{\text{CD}5\text{L},\text{d}}\right)$ to the three patterns of the standard stimuli (IFN/LPS, IL4, and IL10). The criterion of the minimum distance classified 10 of the 12 rCD5L-treated samples (83%) as an IL10-like response and 2 (17%) as an IFN/LPS-like response (Figure 1C, right). Therefore, according to the algorithm, rCD5L promoted a phenotype that resembled that of IL10 in 10 out of 12 donors.

RT-qPCR reinforced these findings, showing that treatment of PB monocytes with rCD5L did not modify *CD80, TNF, CD206*, or *TGM2* expression but did induce a predominant increase in *CD163*, Mer tyrosine kinase (*MERTK*), *CD36*, and vascular endothelial growth factor (*VEGF*) mRNA expression, in a very similar way to IL10 (Figure 1D). We next analyzed the expression of a selected set of these genes in THP1 macrophages and found that IL10 did not modify any of them in a significant manner. Therefore, we assayed the corticosteroid DXM as an additional M2-polarizing stimulus in these cells. THP1 macrophages responded to IFN/LPS, IL4, and DXM by modulating *CD80, TGM2, CD163*, and *MERTK* gene expression in a similar way as PB monocytes (Figure 1E). Interestingly, THP1-CD5L macrophages showed a profile that resembled that of THP1-vector macrophages treated with IL10 or DXM.

Given that STAT3 is the key transcription regulator of IL10 (33), we assessed its activation. Western blot experiments revealed increased STAT3 phosphorylation (Tyr705) in PB monocytes polarized with IL10 and rCD5L when compared with those incubated with control human Alb (Figure 1F, left). Similar results were observed for STAT3 phosphorylation in unstimulated THP1-CD5L vs. control THP1-vector macrophages (Figure 1F, right). Taken together, our data reinforce the notion that CD5L is a molecular driver of M2 macrophage polarization. The data further suggested that THP1 macrophages were suitable for the purposes of the present study.

### CD5L Drives Macrophages to an M2 Functional Phenotype Similar to That Induced by IL10

**Figure 2:**
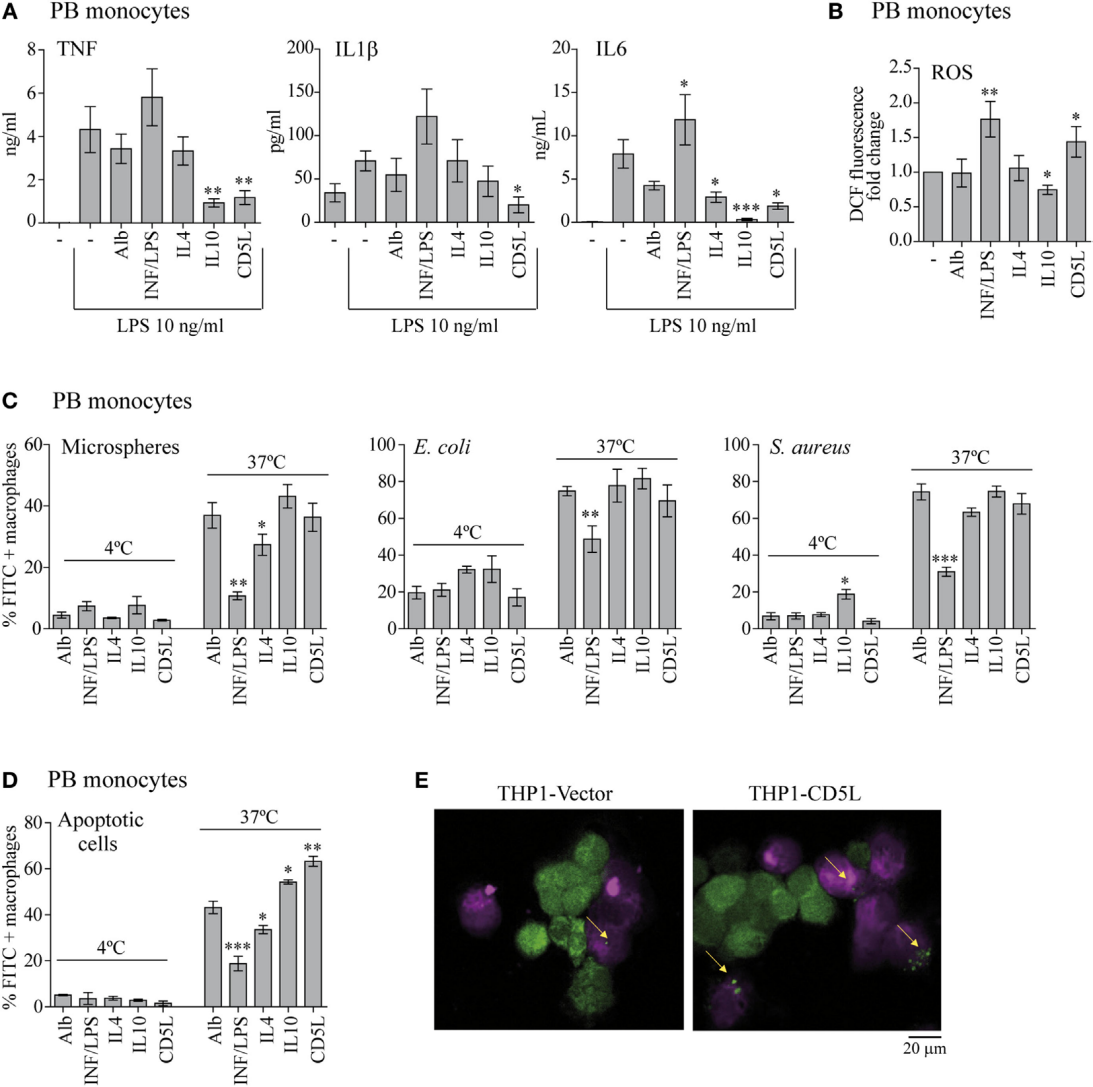
CD5L, like IL10, promotes anti-inflammatory and apoptotic cell pro-phagocytic functions in macrophages. PB monocytes or THP1 macrophages (as indicated) were treated for 72 h with the indicated stimuli or left untreated (−), and the following functional tests were performed. **(A)** The amount of TNF, IL1β, and IL6 was measured by ELISA in culture supernatants of the polarized macrophages, after stimulation with 10 ng/mL LPS for 4 h for TNF, or 24 h for IL1β and IL6. Graphs show mean ± SEM of at least four blood donors, performed in triplicates. **(B)** Intracellular ROS release was quantified *via* the changes in DCF fluorescence. Mean ± SEM of fold change relative to unstimulated cells (−) from three independent experiments performed in triplicate are shown. **(C)** PB monocytes were incubated with 3 µM latex microspheres at a ratio 1:5 (PB monocytes: microspheres), or with *Escherichia coli* or *Staphylococcus aureus* bioparticles both at ratio 1:10 (PB monocytes: bacteria) for 1 h at the indicated temperatures. The percentage of FITC-positive cells was determined by flow cytometry. Data show the mean ± SEM of six independent donors. **(D)** PB monocytes were polarized for 72 h with the indicated stimuli, deep red-stained, and incubated with CFSE-stained apoptotic HepG2 cells at a ratio 1:2 (PB monocytes: apoptotic cells) for 1 h at the indicated temperatures. The percentage of CFSE-positive PB monocytes was determined by flow cytometry. Graph shows the mean ± SEM of five independent experiments. **(E)** Fluorescence microscopy images of unstimulated deep red-stained THP1-vector and THP1-CD5L macrophages (purple) co-cultured with apoptotic CFSE-HepG2 cells (green) for 1 h at 37°C. Data are presented as mean ± SEM. *P ≤ 0.05; **P ≤ 0.01; ***P ≤ 0.001 Student’s *t*-test, vs. macrophages cultured in medium alone (−) column.

Next, we performed a series of functional experiments to determine whether rCD5L-polarized PB monocytes (M-CD5L) share M-IL10 or M-DXM effector mechanisms. In this regard, like M-IL10, M-CD5L secreted lower levels of inflammatory mediators TNF, IL1β, and IL6 in response to LPS, thereby suggesting a decrease in their inflammatory response to this molecule (Figure 2A). In addition, IL10 treatment reduced inflammatory ROS production. Interestingly, treatment with rCD5L had the opposite effect, slightly increasing ROS levels in macrophages (Figure 2B). We studied three additional functional features of macrophages, namely the phagocytosis of latex beads and bacteria, as well as apoptotic cell clearance. In these assays, the percentage of FITC-positive cells increased in a dose-dependent manner when the experiments were performed at 37°C but not at 4°C (Figure S1 in Supplementary Material). These observations indicate that increases in fluorescence were due to uptake rather than to adherence to the cell surface. Interestingly, unlike the inhibitory effect of IFN/LPS, treatment with IL4, IL10, or rCD5L did not alter the phagocytosis of latex beads, Gram-negative *E. coli* or gram-positive *S. aureus* (Figure 2C). On the contrary, when we analyzed the efferocytic ability of rCD5L-polarized macrophages we observed that they responded with increased phagocytosis of apoptotic HepG2 cells. In this regard, rCD5L and IL10 treatments increased the population of FITC-positive cells by 46 and 25%, respectively, when compared with macrophages treated with the control protein Alb (*P* = 0.008 and *P* = 0.0011 Student’s *t*-test, respectively) (Figure 2D). Similar results were obtained when we compared THP1-CD5L and THP1-vector macrophages (Figure 2E; Figure S1 in Supplementary Material). Altogether, the data suggest that CD5L drives macrophages to an anti-inflammatory and high-efferocytic functional phenotype, like that shown by M2, M-IL10.

### CD5L Expression Is Promoted by M2-Polarizing Stimuli

**Figure 3:**
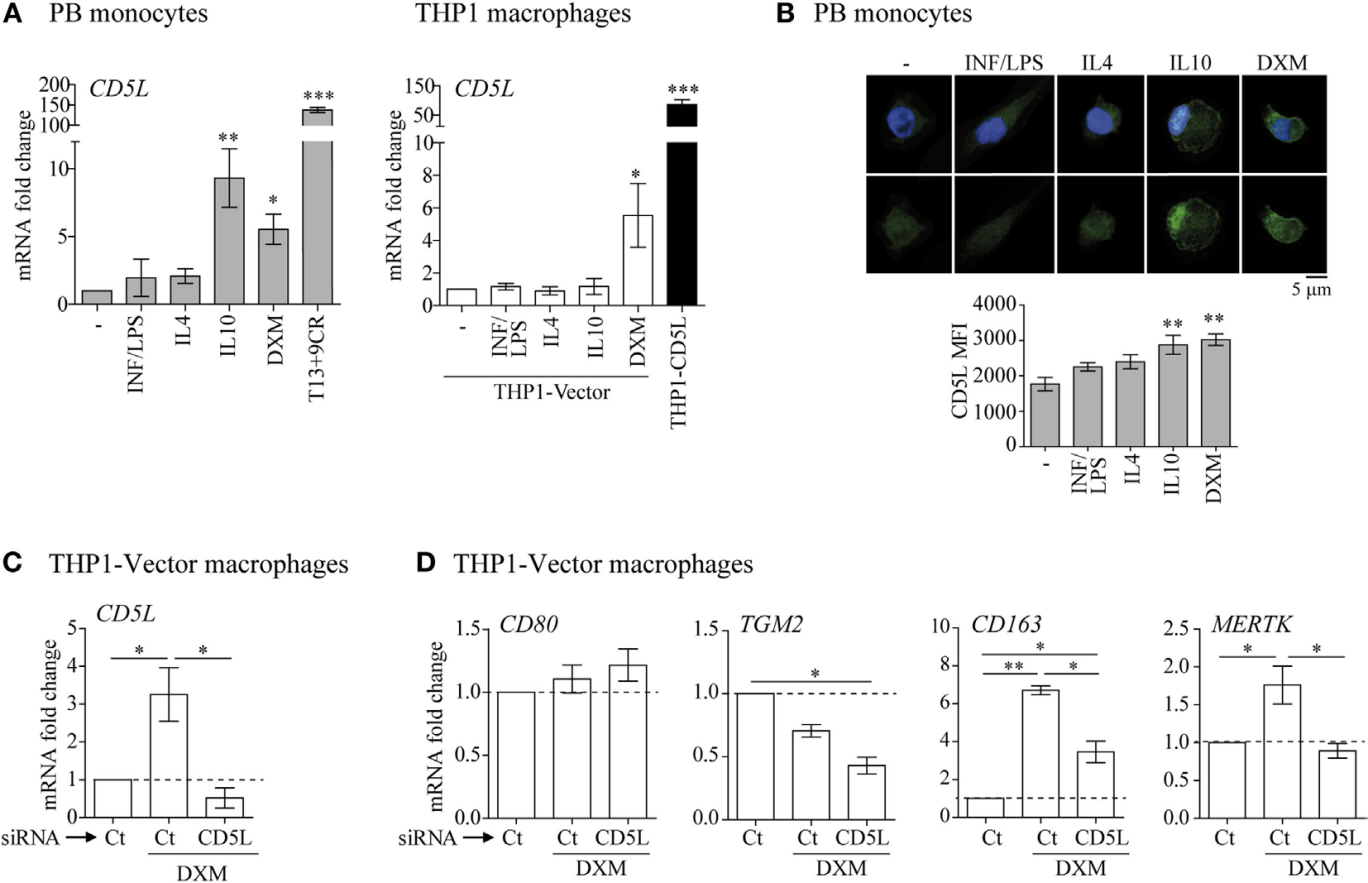
CD5L is expressed in M2 macrophages. **(A)** RT-qPCR analysis of *CD5L* expression in primary macrophages (left) and THP1 macrophages treated with the indicated stimuli for 24 h. Data show mean values of at least five blood donors or five independent experiments. **(B)** IF representative images of CD5L (green) in primary macrophages treated with the indicated stimuli for 72 h. Nuclei were stained with Hoechst (blue). Graphs show CD5L mean fluorescence intensity (MFI) ± SEM of more than 50 macrophages scored in random fields. **(C)** Analysis of *CD5L* mRNA levels in THP1-vector or THP1-CD5L macrophages after transfection with siRNA targeting CD5L (*CD5L*) or a non-targeting negative control (Ct) and 24 h treatment with dexamethasone (DXM). Data show mean values of four independent experiments. **(D)** Relative amounts of mRNA encoding *CD80, TGM2, CD163*, and Mer tyrosine kinase (*MERTK*) measured by RT-qPCR in *CD5L*-silenced (*CD5L*) or non-targeting negative Ct transfected THP1-CD5L macrophages after 24 h of DXM treatment. Data show mean ± SEM of four independent experiments. Data are presented as mean ± SEM. *P ≤ 0.05; **P ≤ 0.01; ***P ≤ 0.001 Student’s *t*-test.

To determine the expression of CD5L in polarized macrophages, we analyzed CD5L mRNA and protein in PB monocytes polarized with INF/LPS, IL4, IL10, or DXM. LXR/RXR synthetic ligands (T13+9CR) were used as a positive control of CD5L upregulation, because these nuclear receptors induce CD5L expression (19, 34). RT-qPCR data showed that CD5L mRNA levels were upregulated 9.31- and 5.54-fold by IL10 and DXM polarization, respectively, when compared with medium alone (Figure 3A, left). Accordingly, DXM-treated THP1-vector macrophages showed a 5.53-fold upregulation of CD5L mRNA (Figure 3A, right). The upregulation of CD5L gene expression was also associated with an increase in protein expression, as observed by immunofluorescence staining and confocal microscopy using an anti-CD5L moAb (Figure 3B). To study the participation of CD5L in the acquisition of the M2 phenotype, we silenced its expression in DXM-treated THP1-vector macrophages. CD5L mRNA induction by DXM in these cells was abolished by siRNA transfection when compared with THP1-vector macrophages transfected with control siRNA (Figure 3C). Interestingly, this abolition was concomitant with a diminished expression of M2 markers CD163 (by 56.8%) and MERTK (by 100%) (Figure 3D). In contrast, the expression of M1 marker CD80 remained unaltered, thereby suggesting that these changes were specific. Altogether, these data indicate that CD5L is expressed in M2 macrophages, and they reinforce the notion that CD5L contributes to the acquisition of an M2 phenotype.

### Autophagy Protein ATG7 Is Involved in M-CD5L Polarization

**Figure 4:**
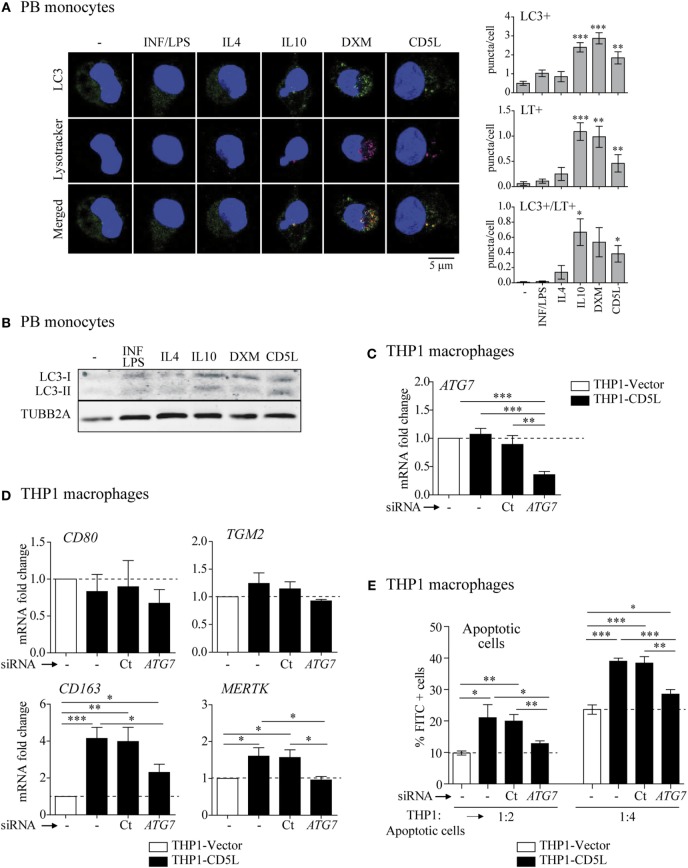
Involvement of autophagy in M-CD5L polarization. **(A)** 72-h treated PB monocytes were stained with a specific LC3 antibody (green), acidic organelles with Lysotracker Red (purple), and nuclei with Hoechst dye (blue). Left panel: representative confocal microscopy images showing LC3 and LysoTracker Red staining and colocalization in yellow (merged). Right graphs: mean ± SEM quantitative data showing the number of LC3 puncta per cell (LC3^+^ puncta/cell) Lysotracker puncta per cell (LT^+^ puncta/cell) and LC3-LysoTracker Red colocalized puncta per cell (LC3^+^ LT^+^ puncta/cell) for four blood donors, including at least 50 cells scored in random fields. **(B)** Immuno-blot of LC3I and II levels in 72-h treated PB monocytes. Representative blots for three independent experiments. Detection of TUBB2A was used as a measure of equal loading. **(C)** Analysis of *ATG7* mRNA levels in THP1-vector or THP1-CD5L macrophages, untreated (−) or after transfection with siRNA targeting ATG7 (*ATG7*) or a non-targeting negative control (Ct). Data show mean values of five independent experiments. **(D)** Relative amounts of mRNA encoding *CD80, TGM2, CD163*, and Mer tyrosine kinase (*MERTK*) measured by RT-qPCR in untreated (−), ATG7-silenced (*ATG7*), or non-targeting negative control (Ct) transfected THP1-CD5L macrophages. Data show mean values of five independent experiments. **(E)** Determination of degree of efferocytosis in THP1-CD5L macrophages, non-targeting negative control transfected cells (Ct), and *ATG*7-silenced cells co-cultured with apoptotic CFSE-HepG2 cells for 1 h at the indicated temperatures and ratios. Data show the mean ± SEM percentage of FITC-positive cells of five independent experiments, as determined by flow cytometry. *P ≤ 0.05; **P ≤ 0.01; ***P ≤ 0.001 *t*-test in **(A,C–E)**.

Both autophagy and LC3-associated phagocytosis may be involved in macrophage polarization (35–39). As CD5L induces autophagy (23), we next examined whether autophagic machinery is involved in CD5L-driven macrophage polarization. Autophagy vesicle formation and fusion with lysosomes were examined in M-IFN/LPS, M-IL4, M-IL10, M-DXM and M-CD5L (Figures 4A,B). To this end, we measured LC3 puncta per cell and the colocalization of LC3 puncta with acidic organelles, the latter detected with the acidotropic fluorescent dye Lysotracker Red. Polarization with IL10, DXM, and rCD5L triplicated LC3 puncta per cell (2.40 ± 0.24, 2.87 ± 0.29, 2.21 ± 0.47, respectively, vs. 0.66 ± 0.19) and promoted Lysotracker Red colocalization (0.57 ± 0.17, 0.41 ± 0.16, 0.36 ± 0.12 double positive puncta per cell, respectively, vs. 0.008 ± 0.008) when compared with treatment with the control protein Alb. In addition, cells were examined for the microtubule-associated protein 1 light chain 3A/B (LC3)-II status by western blot of total cell lysates (Figure 4B). These assays revealed that, like IL10 and DXM, rCD5L-induced high LC3-II levels, thereby further supporting the notion of increased autophagy-dependent mechanisms.

We next silenced the expression of ATG7, an integral component of the cellular autophagic machinery, in THP1-CD5L macrophages and observed phenotypic and functional consequences. In these experiments, siRNA treatment led to ~60% silencing of *ATG7*, as previously reported (23) (Figure 4C). Silencing *ATG7* inhibited *CD163* and *MERTK* mRNAs by 55 and 100%, respectively, when compared with their expression levels in THP1-vector macrophages, but did not affect the expression of M1 marker *CD80* or *TGM2* (Figure 4D). Moreover, ATG7 silencing reduced the efferocytic capacity of THP1-CD5L macrophages by 68% when compared with cells treated with control non-targeting siRNA and using THP1-vector macrophage activity as a reference (Figure 4E). These data suggest that autophagic mechanisms participate in the CD5L-induced M2 macrophage phenotype and function.

### Gene Expression Profile Analysis of CD5L-Expressing Macrophages Reveals ID3 As a Molecular Target

**Figure 5:**
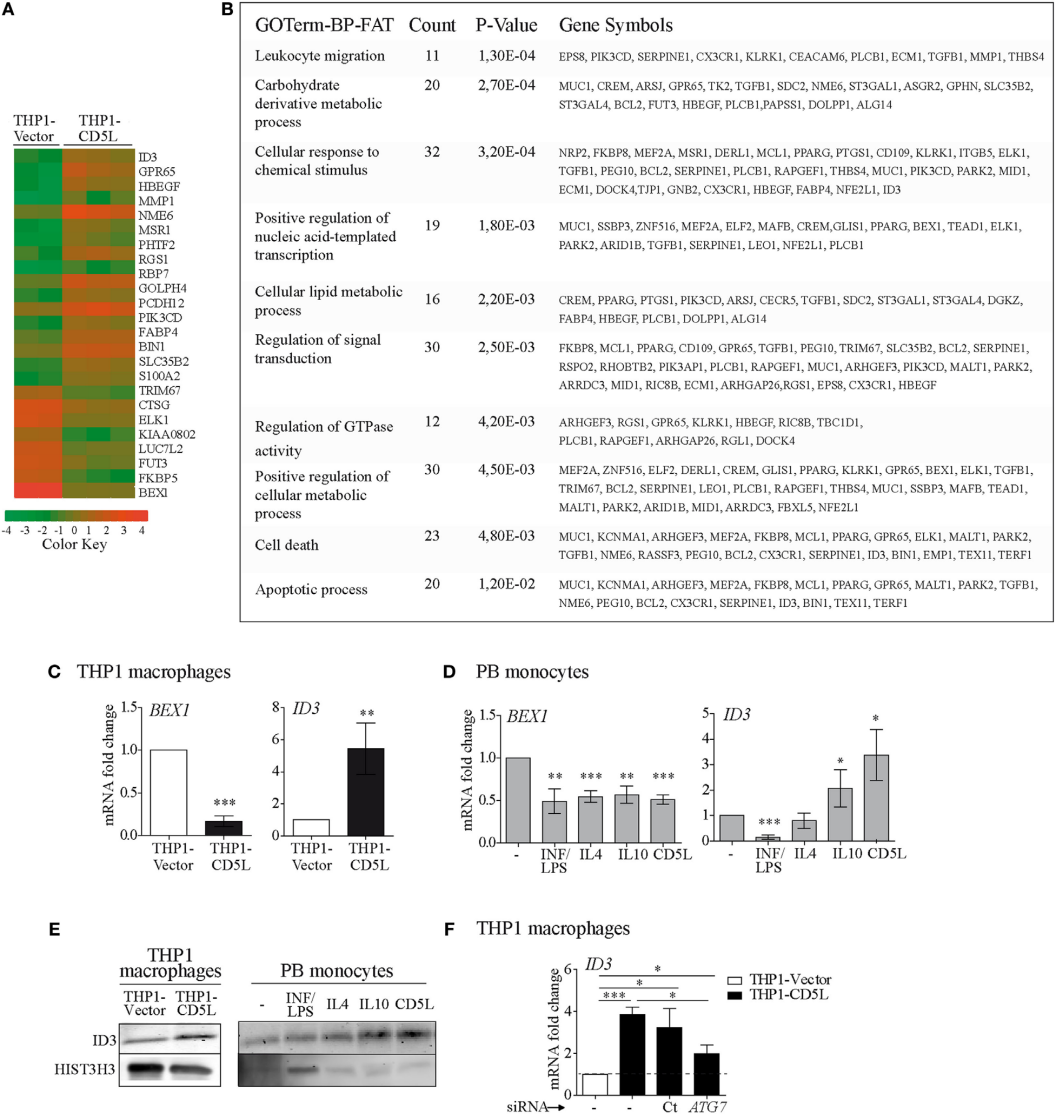
Gene expression profiling analysis of THP1-vector and THP1-CD5L macrophages. **(A)** Global effects of CD5L overexpression on the THP1 cell line. THP1-vector (*n* = 2 replicates) or THP1-CD5L (*n* = 3 replicates). Heat-map of genes up- or downregulated by CD5L expression (>2-fold induction, *P* < 0.05). Positive (orange) and negative (green) changes in gene expression over control cells are shown. **(B)** Gene Ontology analysis of the list of genes modulated by CD5L shows significant enrichment of several biological processes. **(C)** RT-qPCR analysis of brain expressed X-linked 1 (*BEX1*) and *ID3* expression in THP1-vector and THP1-CD5L macrophages. Data show mean values of six independent experiments. **(D)** RT-qPCR analysis of *BEX1* and *ID3* expression in PB monocytes incubated with the indicated stimuli for 24 h. Data show mean values of five blood donors. **(E)** Western blot of ID3 in THP1-vector and THP1-CD5L cells (left) and in 72 h-polarized PB monocytes (right). Representative blot for three independent experiments. Detection of HIST3H3 was used as a measure of equal loading. **(F)** Levels of *BEX1* and *ID3* mRNA in untreated (−) or non-targeting siRNA negative control transfected cells (Ct) and *ATG*7-silenced (*ATG7*) THP1 cells. Data show mean of five independent experiments. *P ≤ 0.05; **P ≤ 0.01; ***P ≤ 0.001 Student’s *t*-test.

Array-based expression profile experiments were performed to compare THP1-vector and THP1-CD5L macrophages. The expression of 16 and 9 genes was upregulated and downregulated, respectively, by CD5L expression (>2-fold induction, *P* < 0.01) (Figure 5A). The list of genes modified by CD5L (>2-fold, *P* < 0.05) was subjected to GO analysis. The use of DAVID bioinformatics resources showed statistically significant enrichment of several functional categories, including leukocyte migration, metabolic processes, signal transduction, and apoptosis, among the list of genes modulated by CD5L (Figure 5B). We further analyzed the expression profiling data to identify an intracellular player for CD5L-mediated macrophage polarization and selected DNA-binding protein inhibitor ID3 (ID3) and the transcription factor brain expressed X-linked 1 (BEX1), which were the most up- and downregulated genes, respectively (fold change vs. THP1-vector: ID3 7.2 and BEX1 −9.59, *P* < 0.0001). Accordingly, RT-qPCR analysis revealed the upregulation of *ID3* and downregulation of *BEX1* in THP1-CD5L vs. THP1-vector macrophages (Figure 5C). Interestingly, *BEX1* mRNA was also downregulated in primary macrophages after all the treatments (i.e., IFN/LPS, IL4, IL10, and rCD5L) when compared with those incubated with medium alone (Figure 5D, left). Therefore, the data indicated no specific involvement of BEX1 in IL10- or CD5L-driven polarization. In contrast, ID3 expression was downregulated in macrophages treated with IFN/LPS, while it was upregulated in those treated with IL10 and rCD5L (Figure 5D, right). We further confirmed CD5L-dependent ID3 upregulation at the protein level by western blot of THP1 macrophage lysates (Figure 5E, left), and in M-IL10 and M-CD5L (Figure 5E, right) when compared with other stimuli or medium alone. Altogether, our data suggest that CD5L and IL10 induce ID3 expression in macrophages.

We then studied whether modulation of *ID3* expression by CD5L was mediated through the induction of autophagy (Figure 5F). Blockade of *ATG7* expression in THP1-CD5L macrophages partially reversed *ID3* mRNA induction (56% inhibition). Taken together, these results indicate that *ID3* mRNA is upregulated in IL10- and CD5L-polarized macrophages and that its expression is regulated, at least in part, by autophagy.

### ID3 Is Involved in M-CD5L Polarization

**Figure 6:**
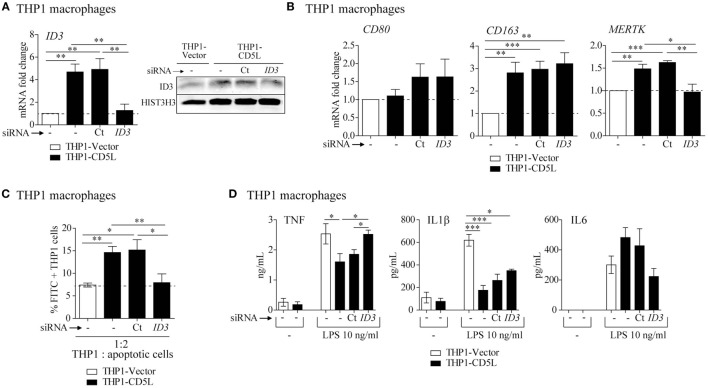
Involvement of transcription factor ID3 in M-CD5L polarization. THP1 macrophages were left untreated (−) or were transfected with siRNA targeting ID3 (*ID3*) or a non-targeting negative control (Ct), and the following functional tests were performed: **(A)** RT-qPCR (left) and western blot representative image (right) of ID3 mRNA and protein silencing. RT-qPCR data show mean of five independent experiments. Detection of HIST3H3 was used as a measure of equal loading in western blot experiments. **(B)** The amount of mRNA encoding *CD80, CD163*, and Mer tyrosine kinase (*MERTK*) was measured by RT-PCR. Data show mean of five independent experiments. **(C)** Analysis of phagocytosis of apoptotic HepG2 cells by flow cytometry. CFSE-apoptotic HepG2 cells were co-cultured with THP1 macrophages for 1 h at a ratio of 1:2 (THP1: apoptotic cells). Graphs show mean ± SEM of five independent experiments. **(D)** Amounts of TNF, IL1β, and IL6 measured by ELISA in culture supernatants after 4 h (for TNF) or 24 h (for IL1β and IL6) of stimulation with 10 ng/mL LPS. Graphs show mean ± SEM of four independent experiments performed in triplicates. *P ≤ 0.05; **P ≤ 0.01, ***P ≤ 0.001; Student’s *t*-test.

ID3 is a transcriptional regulator that negatively controls basic helix−loop-helix transcription factors by forming heterodimers and inhibiting their DNA-binding and transcriptional activity. To determine the contribution of ID3 to CD5L-mediated polarization, we silenced its expression in THP1-CD5L macrophages by siRNA treatment. We observed a ~91% inhibition of *ID3* mRNA (Figure 6A, left) and a reduction of ID3 protein levels in these cells, which leveled out those observed in control cells (THP1-vector) (Figure 6A, right). A decrease in ID3 expression did not modify *CD80* or *CD163* expression, but led to a 100% decrease in *MERTK* mRNA levels, thus completely reversing the effect of CD5L overexpression on THP1 macrophages (Figure 6B). In accordance with a decrease in *MERTK* expression, ID3 silencing abolished the effects of CD5L on the efferocytic activity of these cells by 99% (Figure 6C). Moreover, ID3 silencing in THP1-CD5L macrophages enhanced TNF and IL1β secretion in response to TLR induction, thereby reversing the modulatory effect of CD5L on cytokine secretion in response to LPS (Figure 6D). These data strongly support the notion that the ID3 transcription factor is involved in the induction of the phenotypic and functional characteristics of M-CD5L.

## Discussion

Although macrophage polarization has been characterized in mice through the definition of gene/protein signature markers, secretory profiles, and activation of pathways, the definition of macrophage polarization states in the human is still poorly characterized. Here, we conducted a comprehensive phenotypic and functional profiling of human PB monocytes and THP1 cells treated with standard polarizing stimuli that may be of use for further studies related to macrophage polarization. In this regard, here we prove the utility of our approach to characterize macrophage responses to CD5L. Specifically, we demonstrate that CD5L drives M2 macrophage polarization in a similar manner as IL10. This finding is of relevance because M2 macrophages are key regulators of inflammatory and healing responses, as well as of tumor progression. Identifying novel molecules and mechanisms involved in human macrophage polarization may facilitate the discovery of novel diagnostic and therapeutic tools.

Our polarization studies, which included expression data and mathematical analysis of 5 surface markers in PB monocytes from 26 healthy donors, agree with those of the literature. In addition, they provide with a global picture of phenotypic changes during polarization. Accordingly, we observed that the M1 M-INF/LPS showed upregulation of HLADR in conjunction with the co-stimulatory molecule CD80, which may be indicative of increased antigen presentation (8), and downregulation of CD206, a molecule related to the resolution of inflammation (40). Regarding M2 macrophages, M-IL4 enhanced HLADR and CD80 expression, but also CD206 and CD23, the latter being involved in allergic reactions (41). Surprisingly, IL4, like IFN/LPS, induced the downregulation of CD163, a widely accepted marker of the M2 phenotype (40). In contrast, IL10, which is another M2 stimulus, is upregulated CD163 while it inhibited CD23.

To obtain a global and objective value of these phenotypic changes through which to classify macrophage polarization on the basis of a specific treatment, we developed a mathematical algorithm. For each marker, we defined a unique quantitative descriptor, the *normalized* iMFI magnitude, $\hat{\text{iMFI}}$. This magnitude is independent of the particular fluorescence scale of the different markers assessed. Therefore, the $\hat{\text{iMFI}}$ allows comparison of the effects (up- or downregulation) of the different stimuli on all the markers regardless of their relative amount or fluorescence signal used. We then quantified the distance between the $\hat{\text{iMFI}}$ of the five markers in each donor and each stimuli and compared them with the mean of the profiles by using vectors. This approach, validated by 92% agreement, proved useful to classify the polarized macrophages on the basis of their global phenotype. We believe that this qualifies the method as an excellent tool for classification purposes and that the 8% misclassification detected is probably related to the inherent heterogeneity in humans. The present algorithm allowed us to quantitatively determine that the CD5L-induced macrophage surface marker expression profile is similar to that of the M-IL10 profile. However, the distance values given by CD5L were slightly greater than those produced by IL10, which suggests that the two stimuli led to globally similar but not identical phenotypic responses. In summary, the newly developed algorithm proves useful for determining the effect of different stimuli on macrophage polarization.

Phenotypic changes observed by flow cytometry were complemented by RT-qPCR data. Initial assays confirmed that the changes in CD80, CD206, and CD163 mRNA correlated with those provided by flow cytometry. Therefore, RT-qPCR was used for the analysis of the following additional subset-specific genes: TNF, as one of the main inflammatory cytokines; the multifunctional enzyme transglutaminase 2, MERTK, and the scavenger receptor CD36, all related to phagocytosis and apoptotic cell clearance (40); and VEGF, which is involved in angiogenesis (42). In agreement with previous findings, CD5L modulated these genes in similar way as IL10.

For a better understanding of the effect of polarization treatments on the biological functions of macrophages, we next examined the functional behavior of these cells. In accordance with the literature (7), we observed distinct secretory profiles after LPS stimulation. M-INF/LPS responded to LPS with increased TNF, IL1β, and IL6 expression. In contrast, M-IL10 and also M-CD5L blocked inflammation, producing minimal, or basal levels of these three cytokines in response to LPS. Although CD5L- and IL10-induced macrophage polarization seem to inhibit inflammatory responses to LPS in a similar manner, our data suggest that the effects of CD5L are not caused by the direct induction of IL10 secretion, since rCD5L has no effect on IL10 mRNA or protein levels in macrophages in the absence of TLR stimulation (23). Furthermore, the anti-microbial response involving ROS production was increased in M-CD5L, which contrasts with the diminished levels of ROS detected in M-IL10 (43). These observations thus reinforce the idea that CD5L does not act through direct IL10 induction.

The phagocytosis of pathogens, apoptotic cells, and cell debris is a key feature of macrophage function in host defense and tissue homeostasis. In a set of phagocytosis experiments using latex beads, bacteria, and apoptotic cells, we observed suppressed phagocytic activity by M-INF/LPS. Our findings are in line with reports showing that INF-treated macrophages show impaired phagocytic activity (44–46). Conversely, M-IL10 and M-CD5L showed elevated expression of uptake receptors CD163, CD36, and MERTK, as well as increased efferocytosis, an observation that is consistent with the findings of other studies (47) and that reinforces the role of M2 macrophages in the resolution of inflammation.

Altogether, our phenotypic and functional data suggest that CD5L drives macrophage polarization toward an anti-inflammatory and pro-resolving phenotype. Interestingly, *in vitro*, CD5L expression was restricted to IL10- or DXM-polarized macrophages, thereby pointing to a positive feedback loop between CD5L expression and the maintenance of the M2 phenotype.

An increasing body of evidence shows that the autophagic machinery regulates macrophage polarization (35–39). However, there is discrepancy regarding the contribution of autophagy to M1 and M2 polarization (36, 48, 49). Such discrepancy may be explained by differences in experimental settings and/or in the backgrounds of the macrophages studied. Our data showed increased LC3 puncta and LC3-LysoTracker Red-positive puncta per cell, as well as an increased LC3-II/-I ratio only in macrophages treated with IL10, DXM, and rCD5L, thereby suggesting enhanced autophagy by M2 macrophages. Additional analyses of other proteins that participate in autophagy signaling (e.g., p62/SQSTM1, mTOR, or AMPK) will be necessary to determine the key partners involved. Moreover, regarding the role of autophagy in CD5L polarization, our siRNA experiments targeting ATG7 support the notion that, besides being involved in anti-inflammatory functions (23), autophagy plays a key role in M2 marker expression and also in the clearance of apoptotic cells in M-CD5L.

To identify an intracellular player involved in CD5L-mediated polarization, we performed expression profile experiments comparing THP1-vector and THP1-CD5L macrophages. The THP1 cell line was used because it showed similar results as primary cells regarding marker expression, STAT3 activation, phagocytosis, and efferocytosis. The list of genes modulated by CD5L showed statistically significant enrichment of several functional categories, some previously related to CD5L functions, including leukocyte migration, metabolic processes, signal transduction, and apoptosis (14), thereby validating our data.

Regarding the contribution of ID3 to macrophage polarization, positive feedback between ID3 and M2 polarization has been proposed, because ID3 is upregulated by M2-polarizing stimuli, namely TGFβ (50), lung cancer-conditioned medium, or galectin-1 (51). Our data are in line with these results, because CD5L and IL10, which are also M2 drivers, enhanced ID3 expression. Accordingly, blockade experiments support the notion that ID3 is involved in the anti-inflammatory functions and clearance of apoptotic cells by M-CD5L macrophages. Interestingly, when autophagic flux was blocked by ATG7 silencing in THP1-CD5L cells, ID3 upregulation was partially reversed, thus suggesting that the upregulation of ID3 is autophagy-dependent. To the best of our knowledge, this is the first study to establish a link between ID3 transcription factor and autophagy.

In summary, here we report on a comprehensive method for analyzing human macrophage polarization. This approach has revealed a novel role of CD5L as a driver of M2 macrophage polarization through the upregulation of ID3 and autophagic mechanisms. Our results point to CD5L as a potential target for future therapies seeking to alter the macrophage polarization state. This could be applied in settings such as cancer, where reprogramming tumor-associated macrophages is a promising mode of treatment.

## Ethics Statement

All studies involving human samples were conducted following the Declaration of Helsinki principles and current legislation on the confidentiality of personal data and were approved by the Human Ethics Committee of the Hospital Universitari Germans Trias i Pujol.

## Author Contributions

LS, GA, ET, and NA performed the experiments. LS, GA, and M-RS designed the experiments. LS and M-RS wrote the manuscript. LS, GA, M-RS, and CA analyzed the data. DL and CP developed the mathematical algorithm. All the authors read, discussed, and agreed with the final version of the manuscript.

## Conflict of Interest Statement

The authors declare that the research was conducted in the absence of any commercial or financial relationships that could be construed as a potential conflict of interest.
