# Supplementary material for: CD5L Promotes M2 Macrophage Polarization through Autophagy-Mediated Upregulation of ID3
Source: Front Immunol. 2018 Mar 12;9:480. doi: 10.3389/fimmu.2018.00480 (PMC5858086; doi:10.3389/fimmu.2018.00480)
Supplement: Supplementary file 1 [file Image_1.PDF]

Supplementary Material

CD5L Promotes M2 Macrophage Polarization Through Autophagy-Mediated Upregulation of ID3

Lucía Sanjurjo, Gemma Aran, Érica Téllez, Núria Amézaga, Carolina Armengol, Daniel López, Clara Prats, Maria-Rosa Sarrias*.

Correspondence: Maria-Rosa Sarrias, mrsarrias@igtp.cat

Supplementary Figures and Tables

1.1 Supplementary Figure

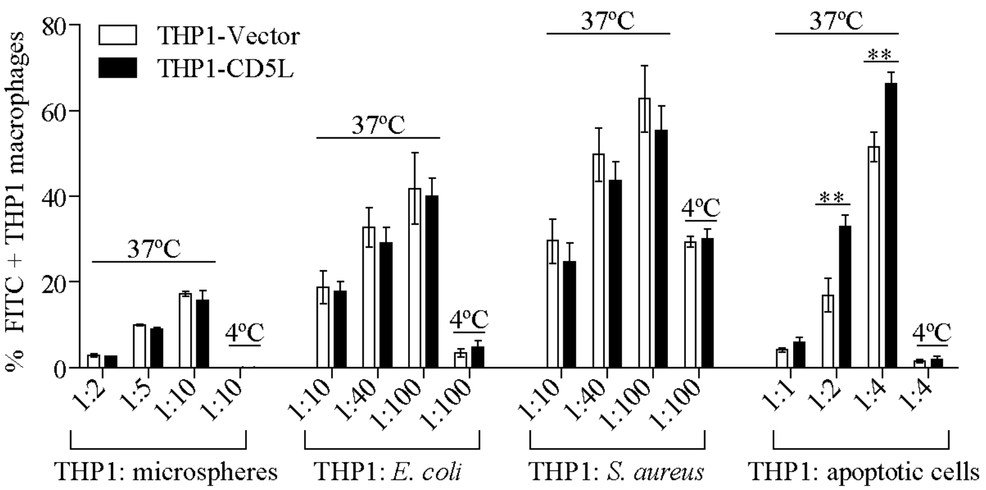

Supplementary Figure 1. Effect of CD5L on THP1 macrophage phagocytosis and efferocytosis activities. THP1-Vector and THP1-CD5L macrophages were incubated with 3-µM latex microspheres, *Escherichia coli* bioparticles, *Staphylococcus aureus* bioparticles, or CFE-apoptotic HepG2 cells for 1 h at the indicated ratios and temperatures. The percentage of FITC-positive cells was measured by flow cytometry. Data show the mean ± SEM of at least three independent experiments. **P ≤ 0.01, Two-way ANOVA.
